# The Evidence for Common Nonsurgical Modalities in Sports Medicine, Part 2: Cupping and Blood Flow Restriction

**DOI:** 10.5435/JAAOSGlobal-D-19-00105

**Published:** 2020-01-03

**Authors:** David P. Trofa, Kyle K. Obana, Carl L. Herndon, Manish S. Noticewala, Robert L. Parisien, Charles A. Popkin, Christopher S. Ahmad

**Affiliations:** From the Department of Orthopaedics, New York Presbyterian, Columbia University Medical Center, New York, NY (Dr. Trofa, Mr. Obana, Dr. Herndon, Dr. Noticewala, Dr. Popkin, and Dr. Ahmad), and the Department of Orthopaedics, Boston Medical Center, Boston University, Boston, MA (Dr. Parisien).

## Abstract

There are a number of nonsurgical modalities used by athletes in attempts to improve performance or prevent, treat, and rehabilitate musculoskeletal injuries. A concise review of available evidence on common nonsurgical modalities used today is necessary, so that practitioners may appropriately counsel patients.

**Methods:**

A comprehensive review of relevant publications regarding cupping and blood flow restriction (BFR) from 2006 through 2019 was completed using PubMed and Google Scholar.

**Results:**

There have been numerous investigations evaluating the efficacy of nonsurgical modalities for a myriad of musculoskeletal conditions. Cupping may be an effective option with low risk in treating nonspecific, musculoskeletal pain. Studies comparing BFR with non-BFR controls suggest that it may increase muscle strength and endurance for individuals undergoing rehabilitation or sport-specific training by mimicking the low oxygen environment during exercise.

**Conclusions:**

Nonsurgical modalities are low-cost treatment strategies with rates of adverse outcomes as low as 0.008% that will likely continue to increase in popularity. Despite the paucity of recent research in cupping and BFR, evidence suggests benefits with use. High-quality studies are needed to effectively evaluate these treatments, so that care providers can provide appropriate guidance based on evidence-based medicine.

The sports medicine specialist enjoys a unique role in the nonsurgical and surgical care of active patients of all skill levels. However, various nonsurgical modalities represent a notable element of sports medicine and musculoskeletal care that is not traditionally taught during orthopaedic training. The increasing use of such modalities merits a review of available evidence to appropriately assess their efficacy and safety, so that clinicians are able to make informed decisions in the care of patients requesting such therapy. In part 1 of this two-part series, we analyze Kinesio taping, sports massage therapy, and acupuncture. Part 2 will discuss cupping and blood flow restriction (BFR) training.

## Cupping

### Background

Cupping is an ancient technique that has been used to treat many disorders from musculoskeletal pain, most commonly of the back, neck, and shoulders, to medical conditions such as hypertension. Recently, there has been an increased interest in cupping, even with elite athletes undergoing treatment as evidenced by the characteristic markings seen on Michael Phelps during the 2016 Olympic Summer Games. When reporting on the use of cupping among elite athletes, the National Broadcasting Company stated that cupping is a “therapy technique that athletes use to help their muscles recover and perform at their best.”^1^ Unfortunately, the validity of this statement has yet to be proven. The following section will review the history, proposed mechanisms of action, and available evidence for cupping in treating musculoskeletal conditions. Given that cupping is an inexpensive and noninvasive modality, it may become an important adjunct treatment in the armamentarium of physicians treating musculoskeletal injuries.

Cupping originated over thousands of years ago and remains a notable component of complementary and alternative medicine in various parts of the world.^2^ Although cupping has been practiced in many cultures, its true origin remains unknown. The earliest known record of cupping dates back to 1550 BC as described in the Egyptian Ebers Papyrus.^3,4^ There is also evidence that cupping, called “Al-hijamah” meaning “to restore to the original size,” was used by ancient Arabic cultures and that it was used to treat hypertension, polycythemia, headaches, and drug intoxication.^5^ The practice later spread throughout the world and was done by physicians, barber surgeons, and bathhouse attendants. Today, it is most commonly used by Eastern medicine practitioners. It was accepted as an official therapeutic practice in Chinese hospitals in 1999 and a 2006 Korean survey found that 93.5% of 6708 physicians used cupping in their practice.^6^

**Figure 1:**
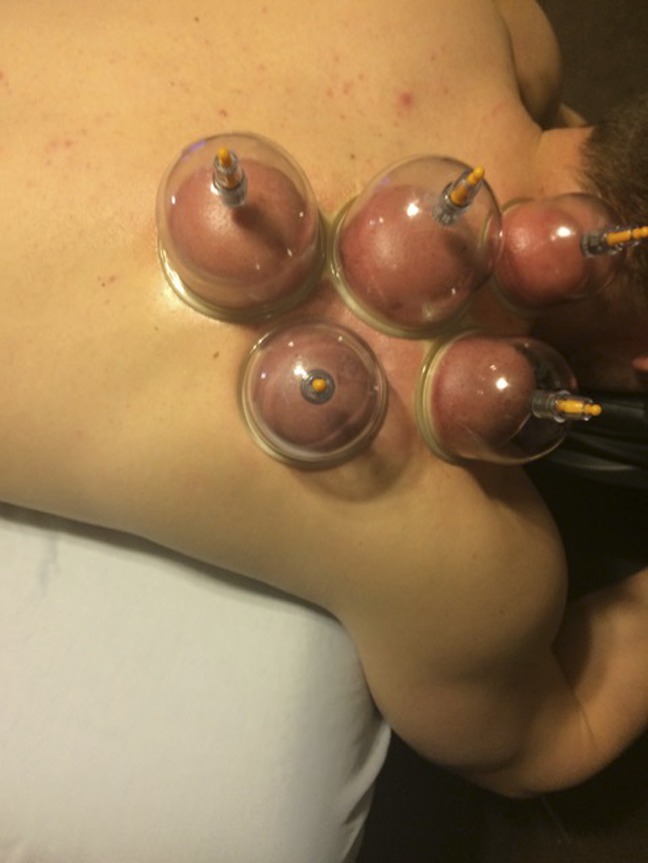
Use of multiple cups on the patients upper back.

**Figure 2:**
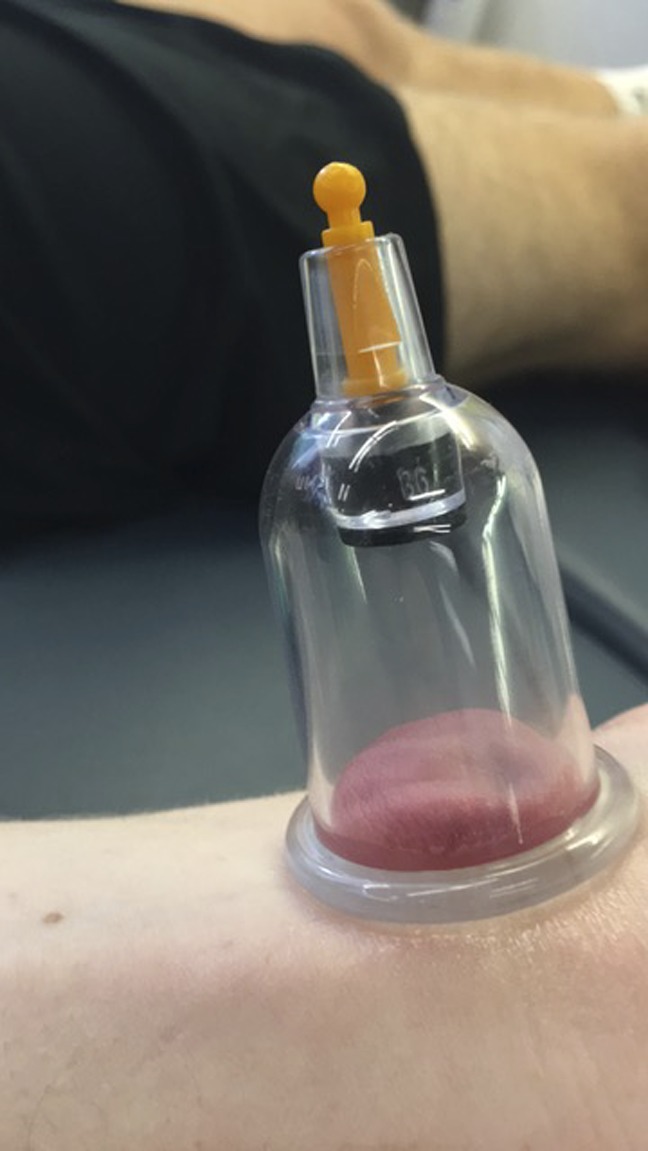
Cup placement on the ventral part of the wrist.

**Figure 3:**
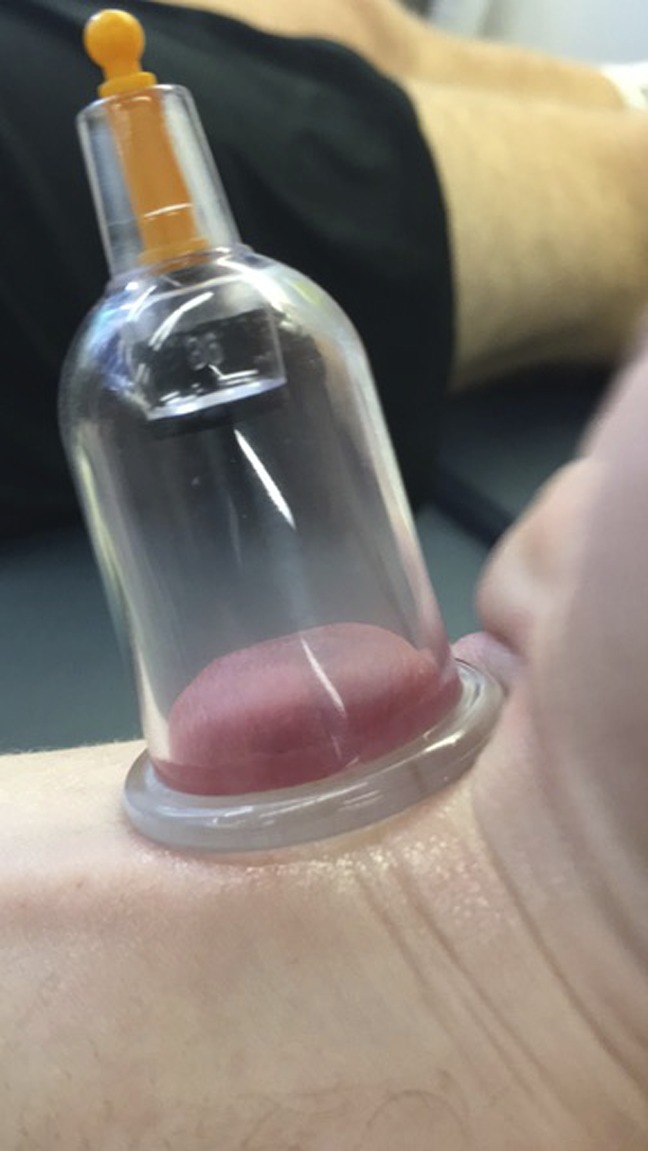
Cup placement on the ventral part of the wrist with flexion.

**Figure 4:**
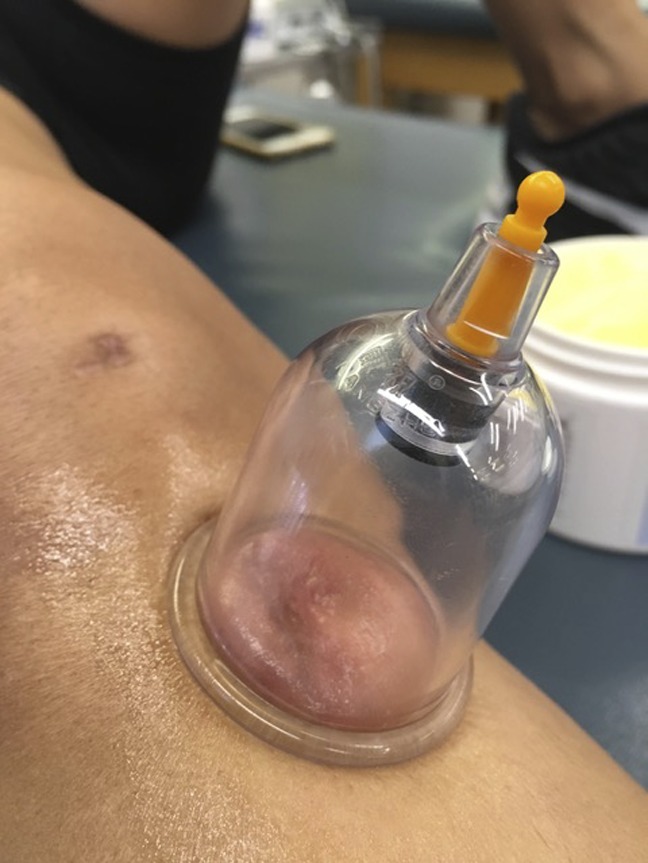
Cup placement distal to the knee.

Various cupping techniques have evolved over time; however, the use of a cup to create a vacuum of negative pressure is common to every method. Cupping is generally done using small round cups of thick glass with a rolled rim to ensure a uniform and airtight contact with skin (Figures 1–4). Negative pressure is created to draw the skin upward by either warming the cup with a flame or by using a manual hand-pump.^39^ Lubricants can also be used allowing movement of the cup over the skin to cover a greater surface area. The most common sites of application are the back, chest, abdomen, and buttocks.^7^ Cupping can be done dry or wet. In wet cupping, the patient undergoes dry cupping for several minutes at which point the suction is abated and a small skin incision is made. Disinfectants are used before making the incision and after cupping is finished to minimize risk of infection.^8^ Suction is then reinitiated to draw out blood that had collected underneath the skin. Finally, cupping results in characteristic skin changes that may include petechiae, erythema, edema, and ecchymosis in a circular pattern that may take days to weeks to resolve.

There have been many different hypotheses and theories regarding the mechanism of action of cupping. Ancient Eastern medicine believed that ailments were a result of alterations or imbalances in one's life force or “Qi” of which cupping was able to correct, similar to theories regarding acupuncture,^9^ while medieval Europeans used cupping to expel evil spirits.^4^ In 1920, Epstein hypothesized that cupping transferred pain from one site to another thus curing the original site of pain, while a psychosomatic theory has been advocated by several research groups more recently proposing the therapeutic effect of cupping is due to a placebo effect alone.^10-12^ Other mechanisms of action of cupping therapy that have been hypothesized include enhancing skin circulation, loosening adhesions and lifting connective tissue, changing the biomechanical properties of skin, altering pressure pain thresholds, adjusting serum P substance levels, stimulating the peripheral nervous system, decreasing oxidative stress, and modulating the immune system.^2,13-15^ In regard to wet cupping, practitioners hypothesize that the expressed blood is rich in harmful toxins that had previously been trapped deeper in the soft tissue.^7,16,17^ Subadi et al. conduced a randomized control trial and found that wet cupping therapy was effective at raising the pain threshold in rats exposed to a hot plate. The authors hypothesized that this effect was attributed to expression of heat shock protein 70 (HSP70) and β-endorphins, both of which mediate pain response.^18^ Despite these findings, a recent extensive review of the literature by Rozenfeld and Kalichman^4^ concluded that that there are “no reliable scientific data clarifying the exact mechanism which can determine the therapeutic effect of cupping.”

What is definitively known about cupping is that the negative pressure results in superficial dermal capillary dilation as a result of the high tensile stress.^9^ In a biomechanical model, Tham et al^9^ illustrated that the soft tissue directly under the rim of the cup compresses while the periphery tenses. The existence of this high tensile stress eventually leads to capillary rupture resulting in the characteristic ecchymosis. Furthermore, cupping is a relaxing treatment modality not unlike massage and acupuncture, and some of its benefits may be a result of stress reduction that is not easily objectified or investigated.

### Evidence

Recently, Chen et al^2^ evaluated the recent research and evidence on the therapeutic efficacy of cupping concluding that there was poor clinical evidence for its use. Despite this, there is an extensive list of clinical uses that cupping has been applied to including cardiovascular disease, angina, migraine, fibromyalgia, hypertension, herpes zoster, Behcet disease, secondary amenorrhea, depression and anxiety, fatigue, metabolic syndrome, and acne vulgaris. However, its most common use is to treat musculoskeletal pain.

Cupping has been investigated for the treatment of nonspecific neck, back, and shoulder pain. Castro Moura et al^19^ conducted a systematic review and meta-analysis on 10 RCT studies analyzing the effectiveness of cupping on back pain. The authors found that cupping therapy reduced quantitative outcomes of cervical and lower back pain in patients compared with controls, who did not receive any treatment. In addition, Lauche et al^20^ investigated dry cupping in an RCT where 50 patients received five cupping treatments over a 2-week period while waiting-list controls did not receive any treatment. The 36-Item Short Form Survey subscales for bodily pain and vitality were markedly improved in patients undergoing cupping, and patients subjectively reported markedly less pain. Wang et al. conducted a meta-analysis on six RCTs comparing cupping therapy with control in management of lower back pain. The authors found a notable decrease in pain and disability in the cupping group.^21^ Kim et al^22^ also conducted a systematic review and meta-analysis on 18 RCTs to analyze cupping therapy on management of general neck pain. The authors found that cupping was effective as either an add-on treatment or by itself. Cramer et al^23^ investigated pulsating pneumatic cupping (a method of cupping that incorporates massage) on chronic neck pain compared with standard medical care in a controlled trial with 50 patients. After 2 weeks with five cupping sessions, patients experienced notable decreases in pain intensity, pain with motion, and functional disability as measured by the neck disability index and improved physical quality of life as measured by the SF-36. Kim et al^24^ compared a heating pad with six sessions of wet and dry cupping in 40 patients done over a 2-week period. Cupping therapy was more effective in improving pain on the numeric pain rating scale and neck function when measured at 3 and 7 weeks of follow-up. Finally, an RCT of 60 patients in Taiwan with chronic neck and shoulder pain were randomized to no intervention or dry cupping.^25^ Patients who received dry cupping had a post-treatment reduction of 6.1 and 5.9 in neck and shoulder pain on a 10-point visual analog scale compared with only 0.2 and 0.6 in controls. No long-term follow-up was provided in this investigation. Despite the fact that these trials may not have been of the highest quality, that various techniques of treatment without standardization were used, and different lengths of follow-up were investigated, the results do suggest that patients may experience decreases in chronic neck, back, and shoulder pain after cupping therapy.

Cupping has also been investigated for knee pain associated with osteoarthritis (OA). Teut et al^26^ have done an RCT in Germany where 40 patients were randomized to eight sessions of dry cupping over 4 weeks or to no intervention. After 4 and 12 weeks of follow-up, there were notable differences found in the Western Ontario and McMaster Universities Osteoarthritis global score, pain intensity on the visual analog scale, and physical component of the SF-36 scale. This led the authors to conclude that pulsatile cupping relieved symptoms of knee OA compared with no intervention. Khan et al^27^ also have done a randomized controlled trial where cupping was found to be as effective to oral acetaminophen in regard to analgesia and decreasing edema related to OA in short-term follow-up.

Finally, cupping therapy has been found to be effective in the management of carpal tunnel syndrome (CTS). In an RCT of 56 hands with CTS, individuals treated with cupping therapy and physiotherapy (transcutaneous electrical nerve stimulation and ultrasonography) experienced notable improvement in severity of CTS and distal sensory latency compared with the control group receiving only physiotherapy.^28^ In addition, in an RCT by Michalsen et al,^29^ patients receiving wet cupping therapy experienced greater improvement in severity of CTS after a single treatment session compared with the control group.

Unfortunately, there are limited data on the use of cupping in treating athletes and its plausible effects on performance. One investigation done by Yang et al^30^ recently studied the use of alternative medicine in the treatment of sports injuries among national Korean volleyball players. In a survey distributed to six team doctors who treated a total of 166 injuries over a single season, cupping was actually one of the least frequently used traditional treatments (7.9%), while acupuncture was the most commonly used (40.4%). However, no data on treatment efficacy were reported.

### Adverse Events

There is variation in the number of adverse events documented related to cupping. These include abscess formation, postinflammatory hyperpigmentation, anemia requiring transfusion, keloids, burns, and blistering.^2,31-33^ In an RCT conducted by Kim et al,^24^ four of 40 participants (10%) experienced adverse events associated with cupping (skin laceration, itching, pain at cupping site, and body ache). Conversely, a cross-sectional survey of residents in Korea by Song et al^34^ found that none of the 45 respondents experienced adverse events associated with cupping therapy. Studies suggest that cupping should not be done greater than 20 minutes to avoid blistering.^4,35^

### Conclusion

There has recently been an increasing interest in cupping and wet cupping among elite athletes and the general cohort. Despite the growing prevalence of cupping therapy, there is a paucity of research, guidance, and regulation particularly regarding its efficacy for improving athletic performance. The available literature may support the use of cupping in treating musculoskeletal pain associated with nonspecific neck, back, and shoulder pain, but there are a lack of recent studies, and trials in the literature are not of high quality. The authors agree with multiple investigations reviewing the use of cupping, in that further investigations are necessary that standardize the therapy used, increase the study cohort, provide sufficient long-term observation, and look at objective end points. Nevertheless, given the available data, the risks associated with properly done dry cupping are low enough that physicians can feel comfortable recommending its use among interested athletes experiencing shoulder, neck, or back pain.

## Blood Flow Restriction

### Background

BFR training (or Kaatsu training) was initially developed in Japan in the mid-1990s.^36^ More recently, BFR training has entered mainstream media because of its prevalent use by professional athletes across a range of sports. The basic premise of BFR is the use of low-load resistance exercise combined with moderate BFR to increase muscle mass and strength, and thereby, enhance sport-specific performance.^37,38^ Typically, a flexible cuff is placed around either the proximal thigh or arm and pressurized to an appropriate level to maintain arterial inflow to the muscle group while occluding venous return during exercise.^39^ The suggested pressure recommendation to achieve maximum anabolic response is approximately 50% arterial occlusion.^40^ Thus, the objective of BFR training is to simulate and receive the benefits of high-intensity resistance exercise while doing low-intensity resistance exercise.^41^ As such, BFR training therapy holds tremendous potential for maximizing athlete strength, endurance, sport-specific performance, and rehabilitation from injury.

Under normal exercise conditions, the increased oxygen demand of skeletal muscle is met by a variety of mechanisms. First, a greater percentage of the cardiac output is routed to the exercising muscle. Second, there is vasodilation of the vascular network of the active muscle with simultaneous vasoconstriction of peripheral parasympathetic vascular beds.^42^ Third, active skeletal muscle produces metabolites such as lactic acid, hydrogen ions, and various other catabolites that invoke multiple anabolic signal transduction pathways leading to skeletal muscle hypertrophy and hyperplasia.^41,43^ Finally, the low oxygen tension in exercising skeletal muscle hastens the recruitment of fast-glycolytic muscle fibers that have high-force–generating capacity.^44^

BFR training attempts to exploit such physiological adaptations of exercise. High-resistance exercise is simulated by extrinsically reducing blood flow to active skeletal muscles during periods of low-resistance exercise (thereby mimicking the low oxygen tension environment in exercising skeletal muscle). A pressurized inflatable cuff or elastic band is placed immediately proximal to the exercising muscle.^36,38,45,^ This constrictive device selectively restricts venous outflow, thereby leading to accumulation of the metabolites, such as lactic acid, in the surrounding capillary network.^41,46,47^ Depending on the objective (rehabilitation versus athletic training), a cuff can be placed either unilaterally or bilaterally.^48^ In effect, the metabolic environment created within the muscle is similar to what the muscle experiences when undergoing high-resistance exercise. The end result is activation of multiple signaling pathways leading to skeletal muscle hypertrophy.

The anabolic potential of BFR training was supported in a study by Takarada et al^49^ evaluating laboratory markers between an experimental cohort doing bilateral knee extension exercises with BFR against a matched-control cohort doing bilateral knee extension exercises with unrestricted resistance. The BFR cohort demonstrated markedly higher levels of lactate and growth hormone; in addition, there was no difference between the cohorts with respect to degradative catabolic markers such as creatine kinase (a measure of muscle breakdown) or lipid peroxide (a measure of oxidative stress).

There can be variability in the physiologic response to BFR training among athletes similar to the variability seen in the physiologic adaptation to high-resistance training seen among athletes.^39,50,51^ For example, the metabolic response to BFR training varies between endurance and sprint runners.^52^ BFR training resulted in higher levels of physiologic stress as evidenced by lower phosphocreatine and intramuscular pH levels in endurance runners compared with sprint runners. These differences are likely the result of pre-existing differences in muscular adaptation that occur during a career of endurance versus sprint running. Quite possibly, sprint runners routinely do under conditions of low intramuscular oxygen tension and thus are not challenged by the metabolic environment created during BFR training. Regardless, the physician and training staff must be aware that different athletes across different sports may have different physiologic responses and adaptations to BFR training.

### Evidence

Several studies have demonstrated increased muscle strength, endurance, and cross-sectional area with low-resistance BFR training.^53-55^ Takarada et al examined the effects of combining resistance training with BFR in elite rugby players in Japan.^33^ Study participants have done 8 weeks of low-load resistance training consisting of bilateral knee extensions with or without BFR. The group that did exercises with BFR demonstrated increased muscle endurance compared with the group without BFR. Furthermore, the group that did exercises with BFR demonstrated an increased cross-sectional muscle area at the end of the study compared with the beginning of the study. Even seasoned athletes have achieved benefits from incorporation of BFR training. Yamanaka et al^55^ had Division IA football players with at least 5 years of resistance training experience do low-load training with and without BFR. The athletes who have done low-load resistance training with BFR demonstrated a markedly greater increase in their 1 repetition maximum for bench press and squats than the group that did not incorporate BFR; furthermore, the football players using BFR demonstrated a markedly greater increase in chest girth.

Lixandrão et al^56^ conducted a systematic review and meta-analysis comparing low-load BFR with high-load BFR in changes to muscle strength and muscle mass. The authors identified 12 studies analyzing muscle strength outcomes between low-load and high-load BFR and 10 studies analyzing muscle mass outcomes between low-load and high-load BFR. The authors found that high-load BFR was superior to low-load BFR in increasing muscle strength, but was comparable with respect to increases in muscle mass.

Multiple studies comparing different training programs have shown low-load BFR resistance training combined with traditional high-load resistance training leads to maximal strength gains.^57-59^ Luebbers et al^57^ had American collegiate football players train 4 days each week for 7 weeks in one of four groups: (1) traditional high-load training, (2) traditional high-load training supplemented with low-load training, (3) traditional high-load training supplemented with low-load BFR training, and (4) modified traditional training supplemented with low-load BFR training. The third group doing traditional high-load training supplemented with low-load BFR training demonstrated the largest increase in squatting 1-repetition maximum. In another similar study evaluating recreational male athletes, high-load unrestricted resistance exercise combined with low-load BFR training was the exercise regimen that led to the greatest increase in maximal isometric elbow extension.^58^

The enhanced muscular adaptations accrued through BFR training have been shown to increase performance in sports-specific tests. Collegiate track and field athletes using low-load BFR training have shown improved 10-meter acceleration and 30-meter sprint times.^60^ Similarly, athletes across a variety of sports who underwent low-load BFR training have shown improved performance in 505 agility and 20-m shuttle-run tests.^53^

Finally, low-load BFR training can be beneficial for athletes during rehabilitation from injury. In patients recovering from anterior cruciate ligament reconstruction, BFR training can attenuate muscle atrophy and enhance muscle development.^61,62^ In an RCT by Cancio et al,^63^ BFR training after closed treatment of distal radius fracture reduced pain with activity and increased self-perceived function compared with the control groups. Hughes et al^64^ conducted a systematic review and meta-analysis on patients with musculoskeletal conditions undergoing low-load BFR training for rehabilitation. They concluded low-load BFR increased strength, but was less effective than heavy-load training. Similarly, Centner et al^65^ also conducted a systematic review and meta-analysis of 11 studies on BFR training in older individuals and found that low-load BFR training had similar effects to non-BFR heavy-load training on muscle hypertrophy, but had lower increases in strength. Loenneke et al^45^ have proposed a progressive model for incorporating BFR training from the early phases of rehabilitation through to the resumption of high-load sport-specific training. This model comprised four sequential phases: (1) BFR alone during periods of immobilization, (2) BFR during low-work rate walking, (3) BFR during low-load resistance exercise, and (4) low-load BFR training combined with normal high-load training.

At present, the widespread adoption of low-load BFR training is hindered by the limited number of high-quality published studies and variability in BFR utilization. Scott et al^39^ in a review of available peer-reviewed English language studies investigating BFR training in the athletic cohort were only able to find 11 investigations that met their inclusion criteria. Studies also demonstrate variability in the BFR stimulus. For example, some investigators use inflatable cuffs to achieve strict control of the BFR stimulus while other studies apply elastic wraps to achieve BFR. The use of elastic wraps for BFR has been demonstrated to provide safe, effective, and valid occlusive stimulus for BFR training.^45,66^ However, the exact pressure applied to limbs is not known when using an elastic wrap. Although strict control of pressures is possible with inflatable cuffs, there is variability among studies in pressures calibrated, and studies do not always report pressures applied. The effect that different BFR pressures have on muscular adaptations is not completely understood.

The literature regarding BFR training in athletes also demonstrates variability in clinical outcomes reported and methodological variability in assessment of outcomes. For example, studies may report limb and/or torso girth measurements as a proxy for muscle hypertrophy.^39,55,57^ However, these measurements can be imprecise. Furthermore, during the course of a variable training regimen over a period of days to months, the distribution and composition of tissues can change, thus confounding measurements.^55^

The current literature has shown that low-load BFR resistance training can increase muscle strength, endurance, and cross-sectional area. However, low-load BFR resistance training produces lower levels of muscle recruitment than high-load resistance training with and without BFR.^56,67,68^ In addition, low-load BFR training does not necessarily increase the strength of the entire musculotendinous unit.

### Adverse Events

A large increase in muscle strength with no corresponding increase in surrounding connective tissue strength may lead to tendon (or connective tissue) injury, especially when athletes attempt to lift heavy loads that can be accommodated by muscles but not tolerated by connective tissues.^39,69^ In addition, nerve compression from excessively high cuff pressure can result in transient numbness and tingling.^70^

Finally, as with any exercise regimen, there is potential risk of injury with BFR training. Only sparse injuries have been reported in the literature in the setting of BFR training.^33^ A case report describes a male ice hockey player recovering from injury with BFR training and being diagnosed with rhabdomyolysis.^71^ A survey of 105 training facilities in Japan reported rhabdomyolysis occurring in 0.008% of BFR trainees.^72^

### Conclusion

Although the body of recent evidence is limited, the current literature suggests that low-load BFR training can increase muscle strength, endurance, cross-sectional area, and potentially sport-specific performance. In addition, low-load BFR training holds promise for enhanced recovery from injury. Finally, for the elite athlete, maximal strength benefits may be achieved by supplementing traditional high-load training with low-load BFR training.
